# Optimal row configuration in jujube-cotton intercropping systems increases cotton yield by enhancing growth characteristics and photosynthetically active radiation in arid region

**DOI:** 10.3389/fpls.2025.1663361

**Published:** 2025-09-26

**Authors:** Jinbin Wang, Peijuan Wang, Xiaofei Li, Zhengjun Cui, Ling Li, Qiang Hu, Hang Qiao, Wei Zhang, Sumei Wan, Guodong Chen

**Affiliations:** ^1^ College of Agriculture, Tarim University, Alar, China; ^2^ Key Laboratory of Genetic Improvement and Efficient Production for Specialty Crops in Arid Southern Xinjiang of Xinjiang Corps, Tarim University, Alar, China; ^3^ State Key Laboratory of Cotton Bio-breeding and Integrated Utilization, Institute of Cotton Research, Chinese Academy of Agricultural Sciences, Anyang, China; ^4^ College of Agriculture, Shihezi University, Shihezi, China

**Keywords:** jujube-cotton intercropping, four rows, photosynthetically active radiation, growth characteristics, total yield

## Abstract

**Background:**

In southern Xinjiang, intercropping cotton with jujube trees improves resource use efficiency and boosts farmers' economic benefits compared to monoculture jujube systems. However, the optimal row configuration for cotton in jujube-cotton intercropping systems remain unclear.

**Methods:**

This study investigated the effects of cotton row configurations [2 rows (IC2), 4 rows (IC4), and 6 rows (IC6)] on cotton growth characteristics, photosynthetically active radiation (PAR), yield, and land equivalent ratio (LER) in jujube-cotton intercropping systems.

**Results:**

The leaf area index (LAI) and leaf area duration (LAD) followed the order of IC6 > IC4 > IC2. The intercepted PAR was improved with the increasing rows of cotton, while the transmitted PAR showed a decreasing trend. Dry matter accumulation (DMA) under IC2 and IC4 decreased by approximately 71% and 36% respectively, compared to IC6. While DMA under IC2 was 54.9% lower than that under IC4. Cotton yield under IC6 increased by approximately 98% and 31% compared to IC2 and IC4, respectively, which demonstrated a 51% significant improvement under IC4 compared to IC2. IC4 and IC6 exhibited a higher LER than IC2. However, the jujube yield under IC6 was lower compared to IC2 and IC4. The total yield under IC4 was higher than that under IC2 and IC6. As the number of cotton rows increased, the rate of improvement in cotton growth characteristics demonstrated a diminishing trend. Cotton yield was significantly correlated with LAI, PAR, and DMA. PAR showed significant relationships with LAI and DMA.

**Conclusion:**

Taken together, four rows' cotton planted between jujube trees is recommended for achieve high crop production in the jujube-cotton intercropping system of South Xinjiang region.

## 1 Introduction

Southern Xinjiang is a typical arid region in China, characterized by abundant sunlight and ample thermal resources. However, its ecologically environment faces severe challenges, including soil desertification, impoverishment, and salinization, which significantly limit sustainable agricultural development in the area (Yu et al., 2022). The jujube tree (*Ziziphus jujuba* Mill.) is a heliophilous species with high light requirements, which exhibits strong adaptability to diverse soil types, tolerancing poor, saline, and alkaline soils (Liu et al., 2020). In recent years, Xinjiang’s jujube cultivation industry has witnessed remarkable growth, emerging as a key pillar of the region’s economy (Li et al., 2023). However, due to the limited availability of arable land (Zhang et al., 2022), expanding the jujube trees cultivation area will inevitably lead to a reduction in the planting area for other crops. Moreover, during the sapling stage of jujube trees (less than 10 years), the jujube yield is relatively low, leading to underutilization of land resources (Wang et al., 2016; Zhang et al., 2019). Cotton (*Gossypium hirsutum* L.) is an salt-alkali tolerant crop, studies demonstrate that intercropping cotton with jujube trees enhances resource use efficiency and productivity (Ai et al., 2021b; Li et al., 2014), while mitigating wind erosion and stabilizing sand (Wang X. et al., 2022), thus promoting sustainable agricultural production.

The jujube-cotton intercropping system represents a primary eco-agroforestry model in southern Xinjiang. This composite system demonstrates remarkable capabilities in optimizing interspecific relationships, improving microclimates, enhancing micro-ecosystems, and boosting economic returns (Wang X. et al., 2022). It plays a vital role in ecological restoration and agricultural development in the arid regions of southern Xinjiang, particularly in areas challenged by saline-alkali soils, sandy winds, and poor soil conditions (Cao et al., 2025; Wang et al., 2017). Jujube-cotton intercropping can reduce evaporation-induced water loss, increase cotton yield, and improve land use efficiency, thereby increasing farmers’ income (Ai et al., 2021b; Wang et al., 2024, 2016). Optimizing crop management measures in jujube-cotton intercropping system synergistically balances productivity, greenhouse gas mitigation, and soil carbon sequestration (Cao et al., 2025). However, In the jujube-cotton intercropping system, the root systems of jujube trees and cotton plants inevitably exhibit ecological niche overlap, leads to competition for nutrients and water, consequently altering nutrient cycling within the system (Ai et al., 2021a; Homulle et al., 2022). Moreover, the canopy overlap between jujube trees and cotton creates competition for photosynthetic characteristics and PAR, which reduces light energy utilization efficiency, ultimately resulting in declined cotton yield (Zhang et al., 2014). Therefore, in the jujube-cotton intercropping system, improper cotton row configuration or inadequate planting density management can intensify intercropping competition.

Optimizing the row spacing configuration and planting density increases the leaf area index, improves PAR distribution within the crop canopy, enhances photosynthetic efficiency, and promotes dry matter accumulation, and achieves high crop yields (Hu et al., 2021; Zuo et al., 2024). Zuo et al. (2024) found that a uniform row spacing configuration of 76 cm with high density optimized the spatial distribution of leaves and bolls, resulting in a higher photosynthetic efficiency and yield. Meanwhile, Gao et al. (2024) showed that a three-row planting pattern under one film (with a row spacing of 76 cm and plant spacing of 7 cm) improved the microenvironment of the cotton canopy and enhanced the light energy utilization rate in the middle and lower layers but did not increase the cotton yield. Zhang et al. (2021) demonstrated that optimizing light energy transmission to the lower canopy enhanced light interception in this region, thereby promoting the development of reproductive structure, thus increased both boll number and weight in the lower canopy, ultimately enhancing yields. However, the optimal light interception rate in intercropping systems is different from with monoculture systems (Mao et al., 2016). Light interception is primarily influenced by row spacing in intercropping cropping systems, followed by plant population density (Mao et al., 2016). Additionally, in jujube-cotton intercropping systems, improper arrangement of cotton planting rows may reduce light energy utilization efficiency due to the shading effect of jujube trees (Zhang et al., 2014). Therefore, the optimal row configuration for cotton in jujube-cotton intercropping systems remain unclear.

We hypothesized that an optimal number of cotton rows would increase leaf area index, reduce canopy light transmittance, and enhance light interception, thereby promoting dry matter accumulation and improving yield. Based on this hypothesis, the main objectives of this study were to investigate the following in the jujube-cotton intercropping system: 1) the effects of row configurations on the leaf area index (LAI) and PAR in cotton; 2) the impacts of row configurations on cotton growth characteristics and yield; and 3) the relationships of cotton yield with growth characteristics and PAR under different row configurations. This study systematically investigated the effects of different row configurations on cotton growth characteristics and yield in jujube-cotton intercropping. The present results provide theoretical and technological support for high-yield and high-efficiency production in the jujube-cotton intercropping system in southern Xinjiang.

## 2 Materials and methods

### 2.1 Site description

**Figure 1:**
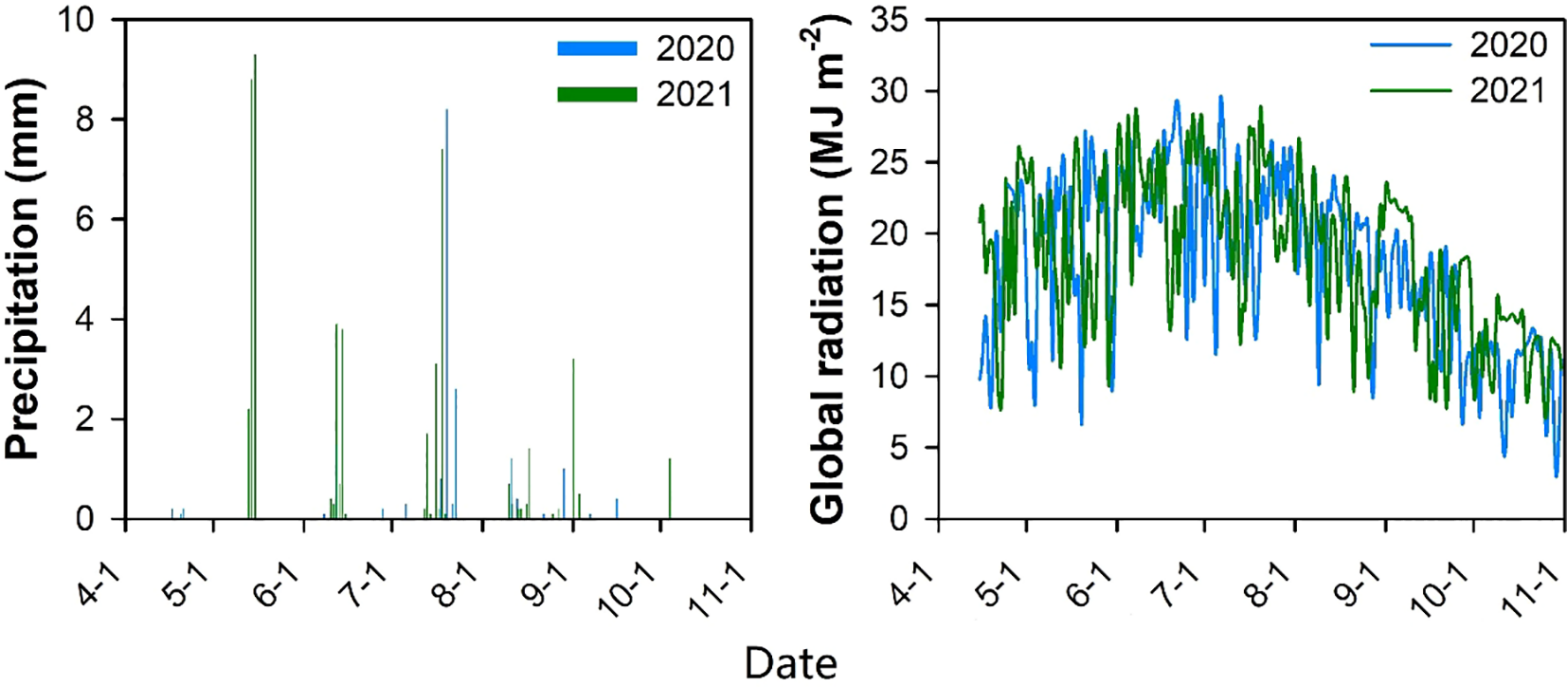
Precipitation and global radiation at experimental site in 2020 and 2021.

This study was conducted in 2020 and 2021 at the Horticultural Experimental Station of Tarim University in Alar, Xinjiang (N40°32’34”, E81°18’07”, elevation 1015 m). The experimental area features a warm temperate extreme continental arid desert climate, with an annual solar radiation ranging from 5594.0 to 6121.2 MJ m^–2^ and annual sunshine duration of 2556.3 to 2991.8 h, corresponding to a sunshine percentage of 58.69%. The frost-free period lasts 180–224 d. Characterized by scarce rainfall, minimal winter snowfall, and intense surface evaporation, the region receives an average annual precipitation of 40.1-82.5 mm and experiences an annual evaporation of 1876.6-2558.9 mm. During the 2020 and 2021 cotton growing seasons, rainfall measured 17.7 and 50.6 mm, respectively, with total solar radiation reaching 3606.8 and 3718.69 MJ m^–2^, respectively (**Figure 1**). The soil properties were as follows: pH 7.90; organic matter content, 11.20 g kg^–1^; total nitrogen, 1.51 g kg^–1^; available phosphorus, 58.70 mg kg^–1^; and available potassium, 107.34 mg kg^–1^.

### 2.2 Experimental design

This study adopted a single-factor randomized block design with five treatments: monoculture jujube (MJ), monoculture cotton (MC), and three intercropping systems of jujube with two rows (IC2), four rows (IC4), and six rows (IC6) of cotton. The experiment included three replicates, with each plot covering an area of 120 m^2^. The experimental jujube orchard was established in 2012 by direct seeding of wild jujube (*Ziziphus jujuba* var. *spinosa*) that was grafted with Huizao jujube (*Ziziphus jujuba* ‘Huizao’) in 2014 and underwent stumping treatment in 2019. The jujube trees were arranged with a planting spacing of 3 m × 1 m. The cotton variety was ‘Tahe No. 2’, with a distance between plants of 11.5 cm, with sowing dates on April 23, 2020, and April 11, 2021. Topping operations were conducted on July 14, 2020, and July 10, 2021, and harvesting occurred on October 23, 2020, and October 17, 2021, respectively. Jujube trees initiate leaf emergence in early May and were harvested in mid-October.

Fertilizer application and irrigation methods involved the setup of drip irrigation tape in both monoculture and intercropping systems, with irrigation and fertilization carried out simultaneously. During the two-year experiment, the fertilization rates and irrigation schedules remained consistent across all crop growth stages. Compound fertilizer (N:P_2_O_5_:K_2_O = 26:13:0) was utilized at a rate of 1305 kg ha^–1^, and over 80% of the water consumed during the crop growth period was supplied through irrigation.

### 2.3 Measurements

#### 2.3.1 LAI and LAD

The LAI of cotton was measured using an LAI 2200C plant canopy analyzer (Li-COR Company of the United States) during the seedling, budding, flowering–boll, and boll opening stages in 2020 and 2021. Based on the LAI, the leaf area duration (LAD) was calculated as follows (Wang et al., 2021):


$$\text{LAD}=\sum_{i=1}^{n}(\frac{{\text{LAI}}_{i+1}+{\text{LAI}}_{i}}{2}\times \text{D})$$

where LAD represents the total leaf area duration of cotton throughout the entire growth period; LAI_i_ and LAI_(i+1)_ denote the LAI of cotton at the (i)-th and (i+1)-th sampling events, respectively; and D indicates the number of days between two consecutive samplings.

#### 2.3.2 PAR, Tr, and IN

In 2020 and 2021, the PAR distribution was measured within different canopy layers of experimental plots using a LI-COR 250A linear quantum sensor at the cotton budding and flowering-boll stages, respectively. Measurements were conducted under clear and windless weather conditions between 12:00 and 13:00 in areas of the plots with uniform growth. The horizontal measurement distance spanned 0–165 cm in all plots, and the vertical measurement distance covered 0–60 cm during the squaring stage and 0–100 cm during the flowering–boll stage. Horizontal measurements were taken at 15-cm intervals from left to right, and vertical measurements at 20-cm intervals from bottom to top.

To address potential errors in PAR measurements caused by transient weather variations during the observation period, which could lead to incomplete synchronization of PAR measurements across treatments, this study employed relative values (transmitted PAR rate (Tr) and intercepted PAR rate (IN)) to mitigate such discrepancies. Tr and IN were calculated using the following equations (Xue et al., 2015; Zhang et al., 2021).


$$\text{Tr}=\frac{{\text{PAR}}_{i}}{{\text{PAR}}_{I}}$$

$$\text{IN}=\frac{\left({\text{PAR}}_{i}-{\text{PAR}}_{i-1}\right)}{{\text{PAR}}_{I}}$$

where PAR_I_ is the incident PAR at the top of the canopy (μmol m^–2^ s^–1^), and PAR_i_ and PAR_i-1_ are the incident PAR at canopy heights i-th and (i-1)-th of the canopy, respectively. At the budding stage, i represents 60, 40, and 20 cm, and i-1 represents 40 cm, 20 cm, and the surface. At the flowering–boll stage, i represents 100, 80, 60, 40, and 20 cm, and i-1 represents 80, 60, 40, and 20 cm and the surface.

#### 2.3.3 Dry matter accumulation and distribution

Dry matter accumulation was determined by the method described by Dai et al. (2015). In 2020 and 2021, five cotton plants were randomly selected from each plot during the seedling, budding, flowering-boll, and boll opening stages. The samples were transported to the laboratory, where they were initially deactivated in a 105°C oven for 30 min and then dried at 80°C until reaching a constant weight. After weighing, the dry matter accumulation was converted to per hectare values. At the cotton boll opening stage, the dry matter accumulation in leaves, stems, bolls, and lint were measured, and the dry matter distribution rate for each organ was calculated.

#### 2.3.4 Crop growth rate and net assimilation rate

The crop growth rate refers to the increase in dry matter weight per unit time (kg ha^−1^ d^−1^). The net assimilation rate represents the dry matter accumulation per unit leaf area during a specific growth period (kg ha^−1^ d^−1^). The specific calculation equations were as follows (Yin et al., 2017):


$$\text{CGR}=\frac{\text{D}2-\text{D}1}{\text{T}2-\text{T}1}$$

$$\text{NAR}=\frac{\text{D}2-\text{D}1}{\text{T}2-\text{T}1}\times \frac{{\text{Ln}}_{L2}-{\text{Ln}}_{L1}}{\text{L}2-\text{L}1}$$

where CGR and NAR represent the crop growth rate and net assimilation rate of cotton, respectively; D1 and D2 denote the dry matter accumulation of cotton during the T1 and T2 stages, respectively; and L1 and L2 indicate the leaf area of cotton at the T1 and T2 stages, respectively.

#### 2.3.5 Yield and LER

Cotton and jujube were harvested by plot at the physiological maturity stage, and the yield was determined, with the final results converted to kg ha^–1^. The total yield in the intercropping system was equal to the sum of the jujube and cotton yields.

The land equivalent ratio (LER) is used to evaluate land productivity in intercropping systems. The specific calculation equation was as follows (Wiley, 1979):


$$\text{LER}=\frac{{\text{Y}}_{\text{IC}}}{{\text{Y}}_{\text{MC}}}+\frac{{\text{Y}}_{\text{IJ}}}{{\text{Y}}_{\text{MJ}}}$$

where YIC and YIJ represent the yields of cotton and jujube, respectively, in the intercropping system, and YMC and YMJ denote the yields of cotton and jujube, respectively, in the monoculture systems. LER > 1 indicates that intercropping has a yield advantage; LER< 1 indicates no intercropping advantage.

### 2.4 Data analysis

Statistical analysis was performed using SPSS 20.0 (SPSS Inc., Chicago, IL, USA) for analysis of variance (ANOVA). The least significant difference (LSD) method at the P< 0.05 level was applied to identify significant differences among treatments. Pearson correlation analysis and principal component analysis were used to evaluate the relationship between cotton yield and LAI, LAD, PAR, dry matter accumulation, growth rate, and net assimilation rate. Figures were plotted using Sigmaplot 12.5 and Origin 21.0.

## 3 Result

### 3.1 Response of LAI and LAD to row configuration

**Figure 2:**
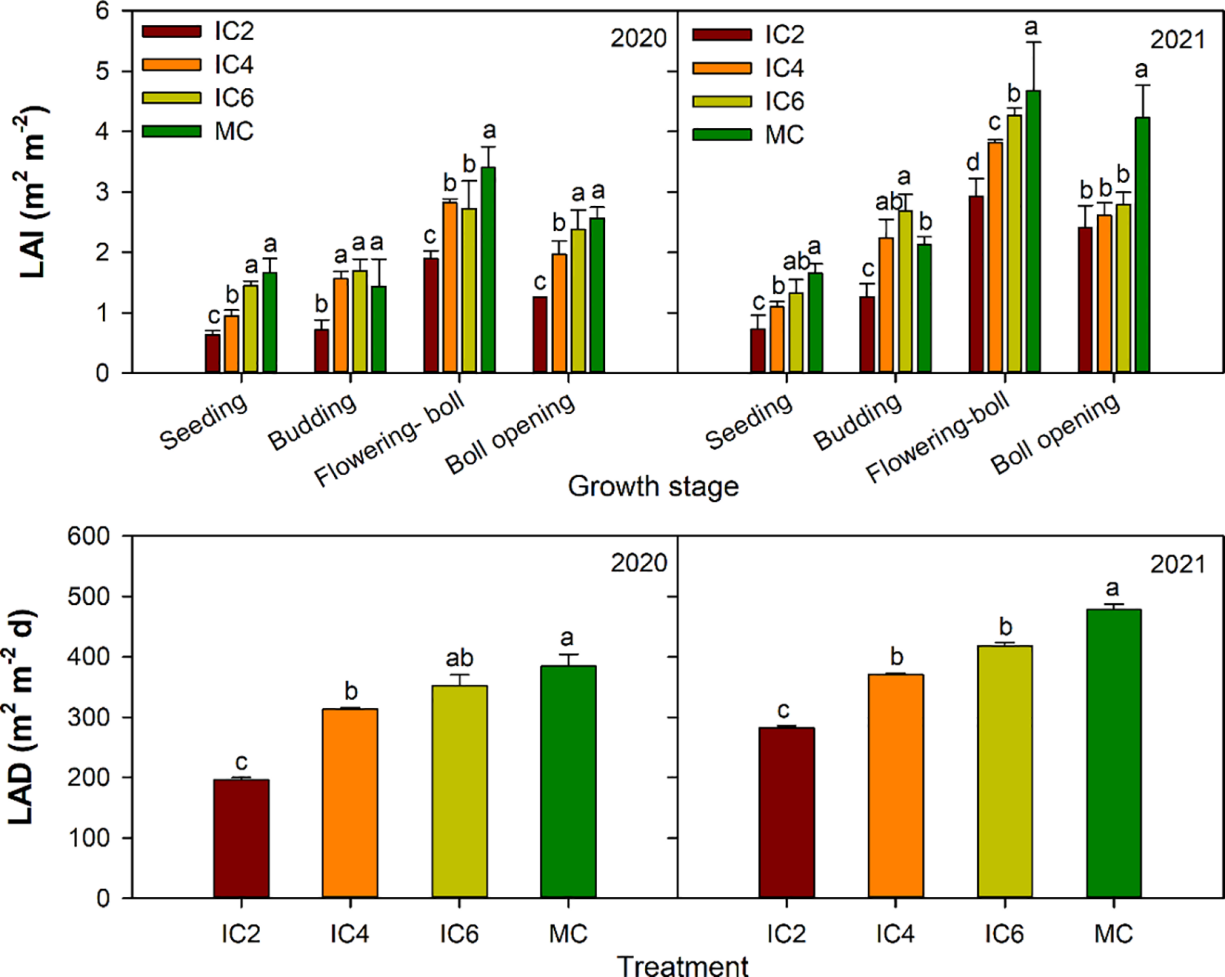
Leaf area index (LAI) and at different growth stages leaf area duration (LAD) under different treatments in 2020 and 2021. IC2, IC4, and IC6 represent jujube intercropped with two, four, and six rows of cotton; MC, monoculture cotton. Different lowercase letters indicate significant differences among treatments at *p<* 0.05.

As cotton developed, LAI initially increased then decreased, peaking at the flowering-boll stage (**Figure 2**). The mean LAI under IC2, IC4, and IC6 decreased by approximately 50%, 22%, and 9%in 2020 and by 42%, 23%, and 13%in 2021, compared to the MC treatment, respectively. In the intercropping system, LAI under IC6 increased by 83% and 13% in 2020 and by 51% and 13% in 2021, compared to IC4 and IC2, respectively. LAI under IC4 improved by 62% in 2020 and 33% in 2021compared to IC2, respectively. The mean LAD under IC2, IC4, and IC6 decreased by 49%, 19%, and 8% in 2020 and by 44%, 28%, and 18%in 2021, compared to MC, respectively. LAD under IC6 increased by 80% and 60% in 2020 and by 45% and 28% in 2021 compared to IC4 and IC2, respectively. LAD under IC4 showed increases of 13% both in 2020 and 2021compared to IC2, respectively. Therefore, as planting rows increased, both the LAI and LAD of cotton rose, though the increments observed between IC2 and IC4 were greater than those between IC4 and IC6.

### 3.2 Response of PAR to the row configuration

**Figure 3:**
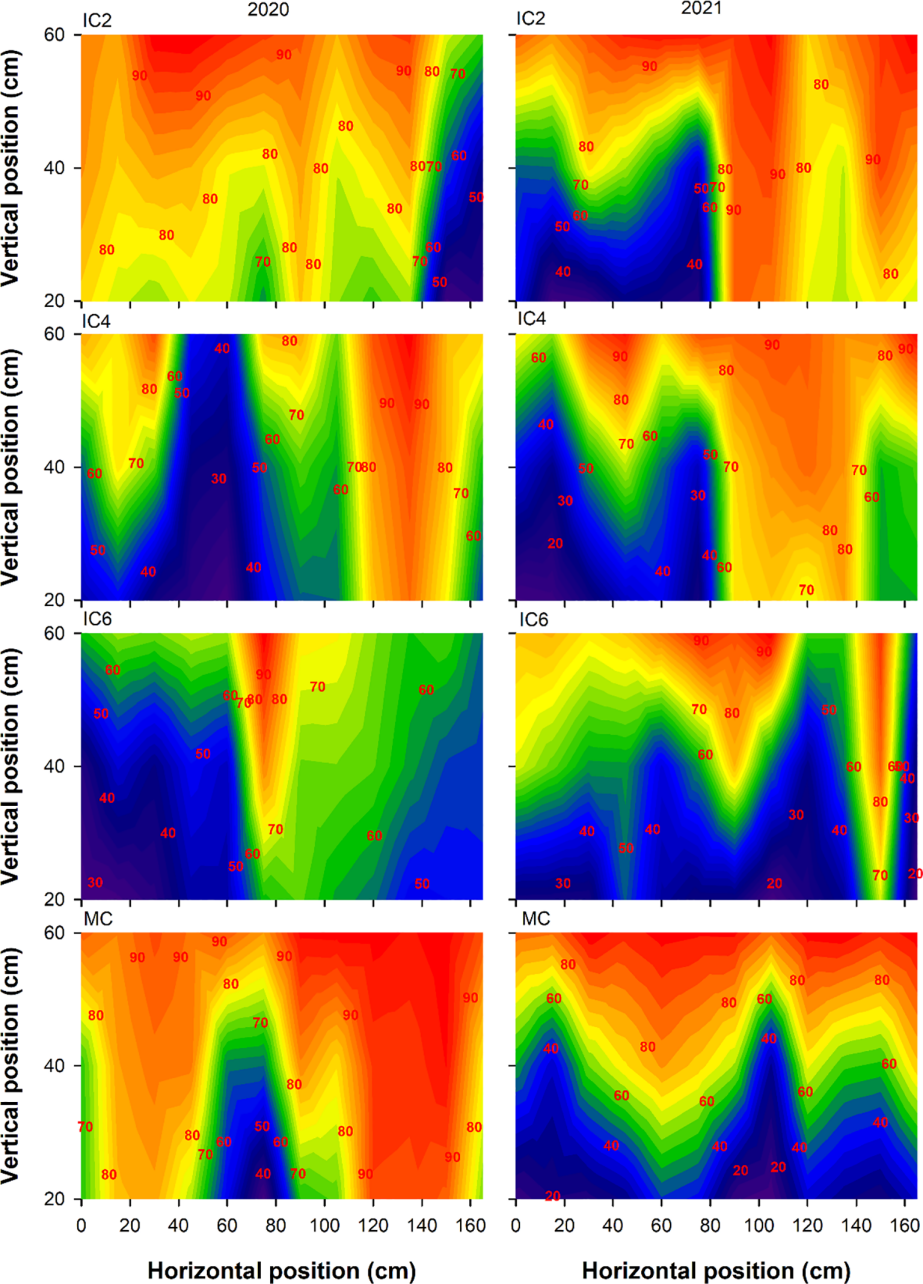
Transmitted PAR rate (%) at the budding stage under different treatments in 2020 and 2021. IC2, IC4, and IC6 represent jujube intercropped with two, four, and six rows of cotton; MC, monoculture cotton.

**Figure 4:**
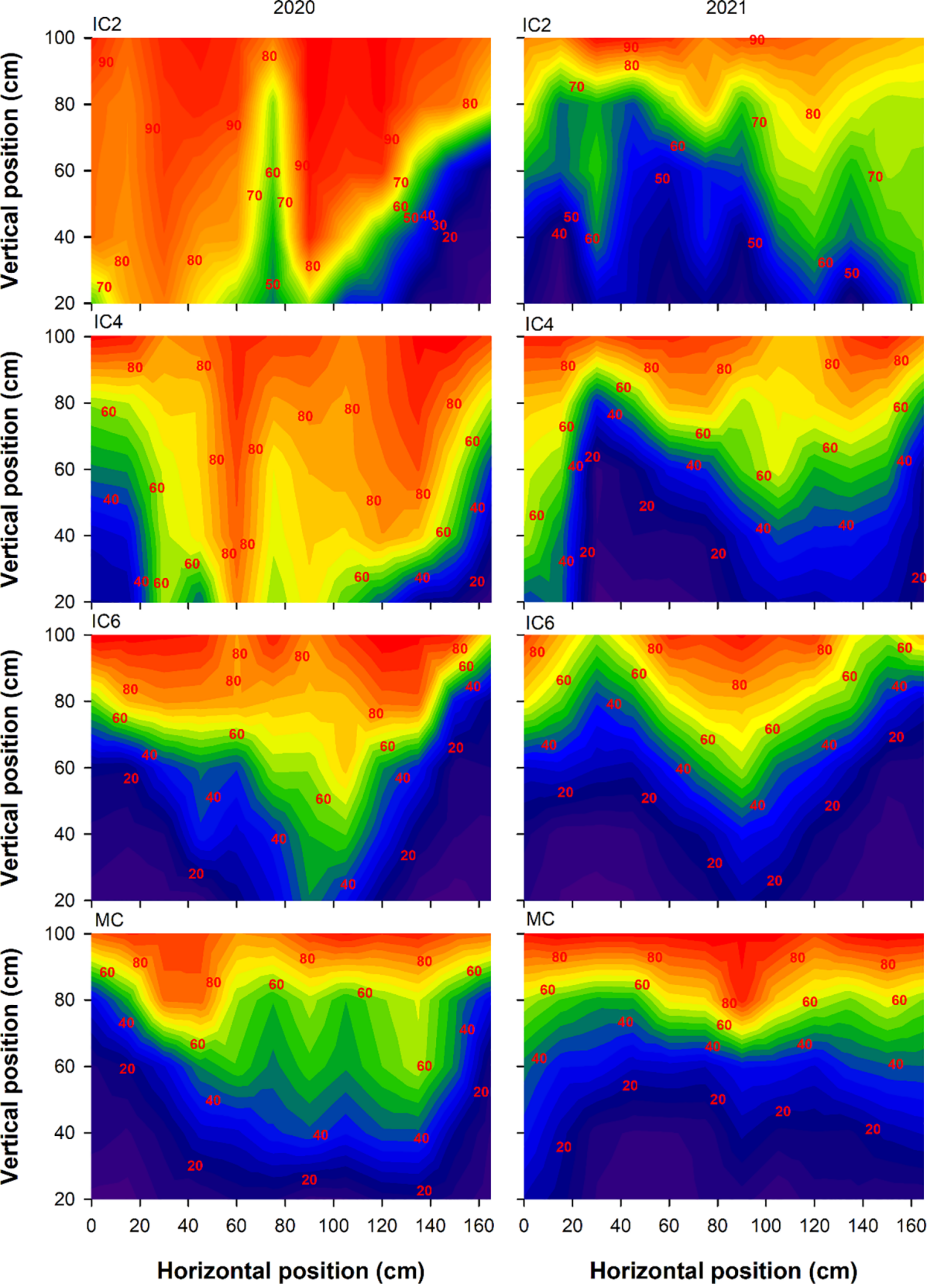
Transmitted PAR rate (%) at the flowering-boll stage under different treatments in 2020 and 2021. IC2, IC4, and IC6 represent jujube intercropped with two, four, and six rows of cotton; MC, monoculture cotton.

Compared to MC, the Tr at the cotton budding stage under IC2, IC4, and IC6 decreased by 5.5%, 24.9%, and 47.1% in 2020, respectively (**Figure 3**), increased by 27.5% and 5.8% under IC2 and IC4 but decreased by 10.0% under IC6 in 2021, respectively. In the intercropping system, the Tr following the pattern of IC2 > IC4 > IC6. Compared to MC, the Tr at the flowering-boll stage under IC2, IC4, and IC6 increased by 61.0%, 47.5%, and 7.1% in 2020, respectively, IC2 and IC4 showed increases of 53.9% and 18.5%, respectively, while IC6 exhibited an 11.5% decrease in 2021(**Figure 4**). Compared to IC6, the Tr under IC2 and IC4 showed increases of 50.3% and 37.6% in 2020, of 73.9% and 33.9% in 2021, respectively. Additionally, the Tr under IC2 was increased by 9.2% in 2020 and 29.9% in 2021, compared to IC4 respectively.

**Figure 5:**
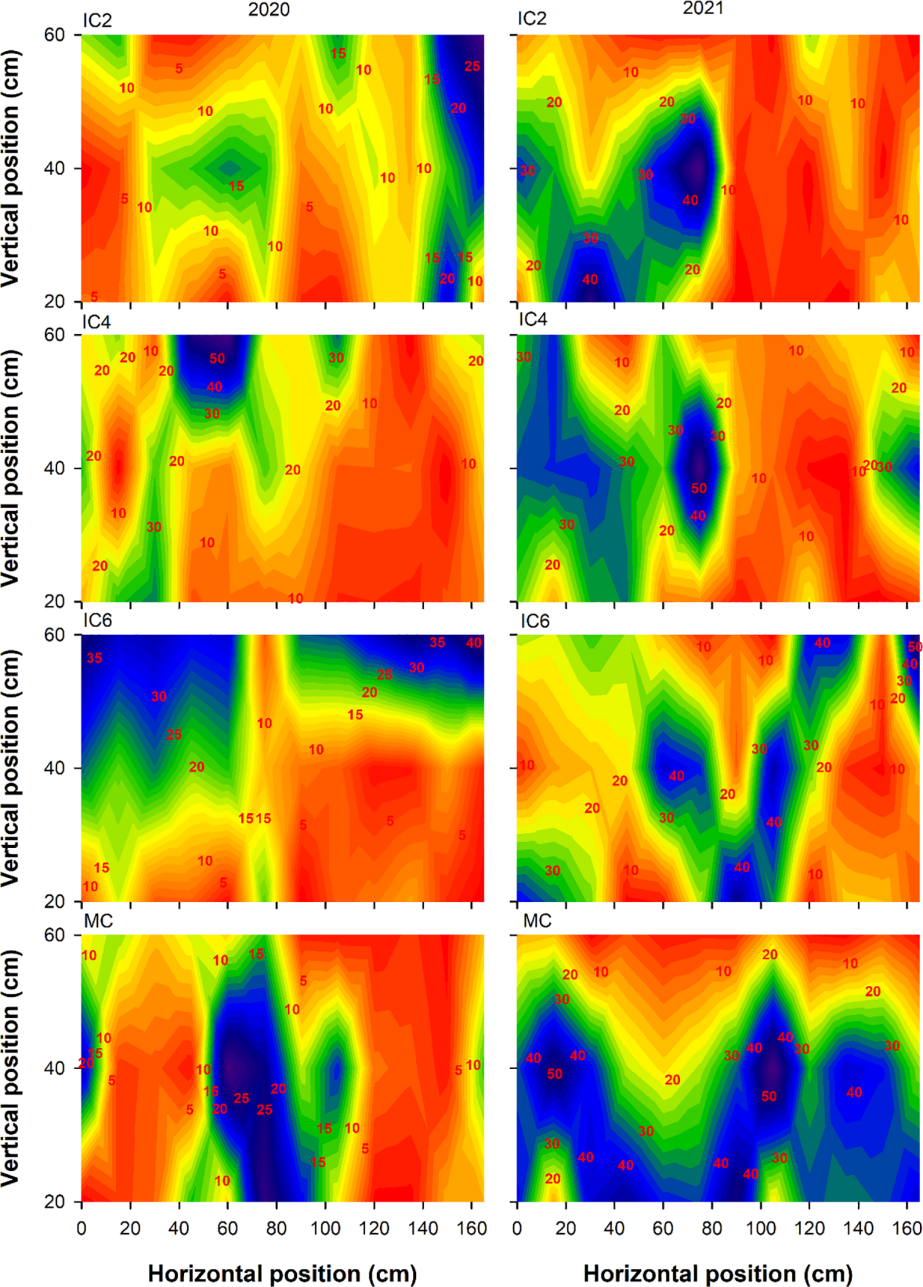
Intercepted PAR rate (%) at the budding stage under different treatments in 2020 and 2021. IC2, IC4, and IC6 represent jujube intercropped with two, four, and six rows of cotton; MC, monoculture cotton.

**Figure 6:**
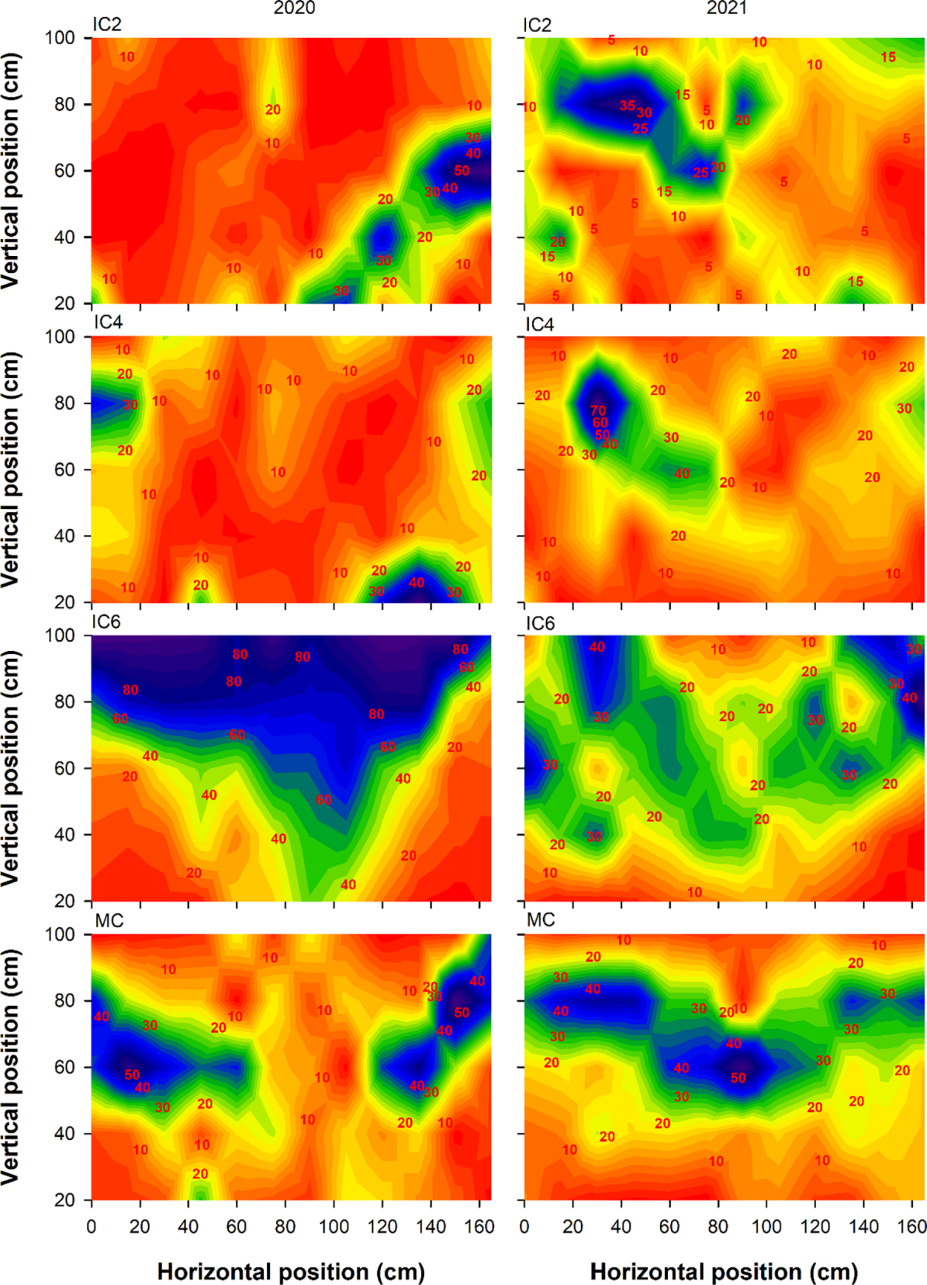
Intercepted PAR rate (%) at the flowering-boll stage under different treatments in 2020 and 2021. IC2, IC4, and IC6 represent jujube intercropped with two, four, and six rows of cotton; MC, monoculture cotton.

IN at the budding stage exhibited the pattern of IC6 > IC4 > IC2 in both 2020 and 2021 (**Figure 5**). The IN under IC6 increased by approximately 77% and 6% in 2020 and by 71% and 26% in 2021 compared to IC2 and IC4, respectively. The IN under IC4 showed increases of 67% in 2020 and 36% in 2021, compared to IC2, respectively. Compared to MC, the IN under IC2, IC4, and IC6 at the flowering-boll stage decreased by 43%, 35%, and 5%in 2020, respectively; these showed reductions of 41%, 10.0%, and −4% in 2021, respectively (**Figure 6**). Compared to IC2 and IC4, the IN under IC6 increased by 66% and 40%in 2020 and by 44% and 13%in 2021, respectively. Additionally, the IN under IC4 demonstrated improvements of 13% in 2020 and 35% in 2021 compared to IC2, respectively.

### 3.3 Response of dry matter accumulation and distribution to the row configuration

**Figure 7:**
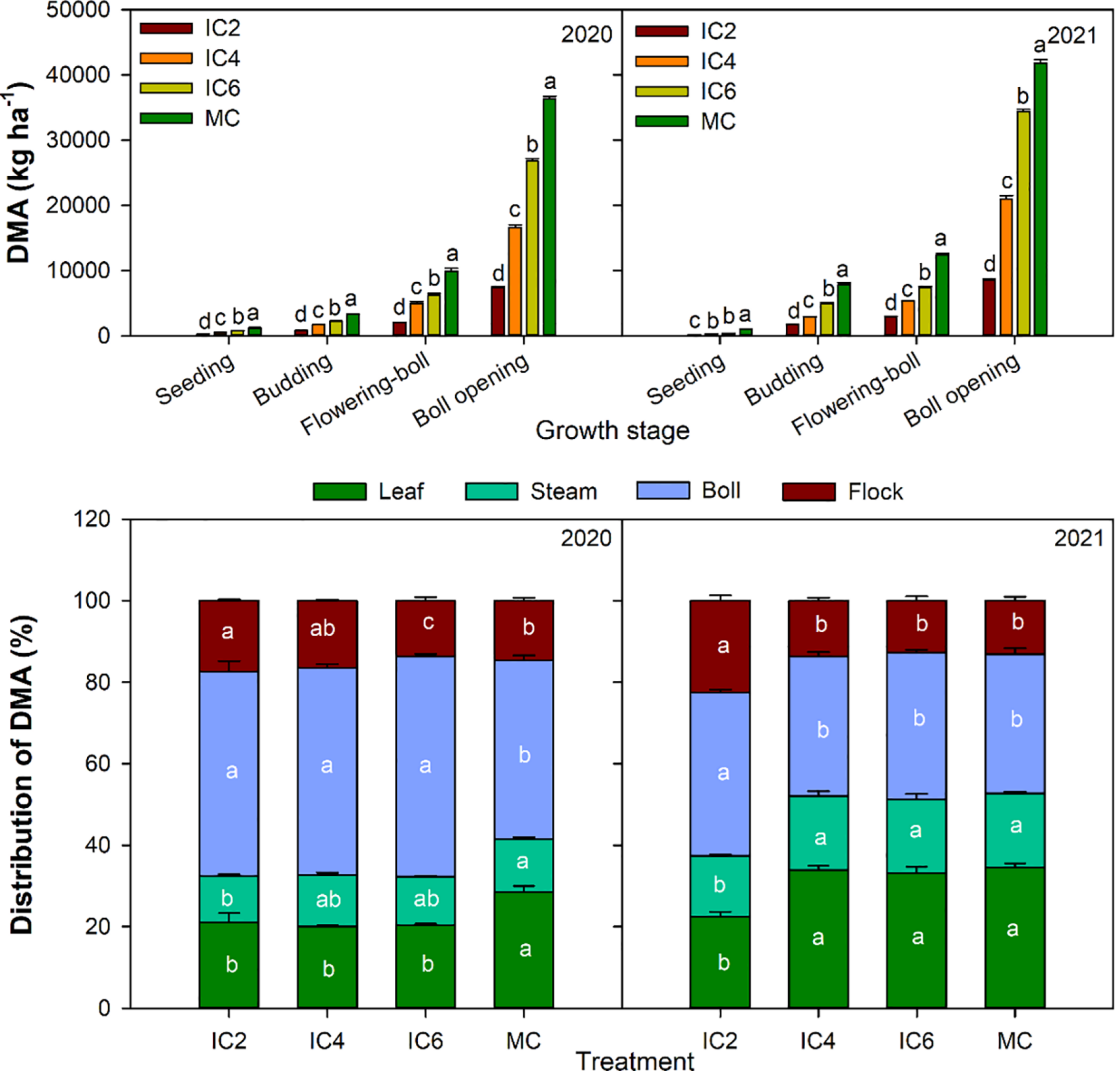
Dry matter accumulation at different growth stages and dry matter distribution at the boll opening stage under different treatments in 2020 and 2021. IC2, IC4, and IC6 represent jujube intercropped with two, four, and six rows of cotton; MC, monoculture cotton. Different lowercase letters indicate significant differences among treatments at *p<* 0.05.

Compared to MC, the mean DMA under IC2, IC4, and IC6 showed significant decreases of 79.0%, 52.0%, and 28.8%, in 2020 and 78.6%, 53.1%, and 25.2%, in 2021, respectively (**Figure 7**). Across both years, the mean DMA under IC2 and IC4 was significantly reduced by 71.0% and 35.6% compared to IC6, under IC2 showed a significant decrease of 54.9% compared to IC4. Compared to MC, IC2, IC4, and IC6 reduced stem and leaf allocation at the boll opening stage of cotton but increased the boll allocation ratio in 2020. In 2021, boll allocation under IC2 significantly increased by 30.5%, 28.4%, and 32.5%, compared to the MC, IC4, and IC6 treatments, respectively. Concurrently, the stem and leaf allocation under IC2 significantly decreased by 28.1%, 27.0%, and 29.1% compared to the MC, IC4, and IC6 treatments, respectively.

### 3.4 Response of the CGR and NAR to the row configuration

**Figure 8:**
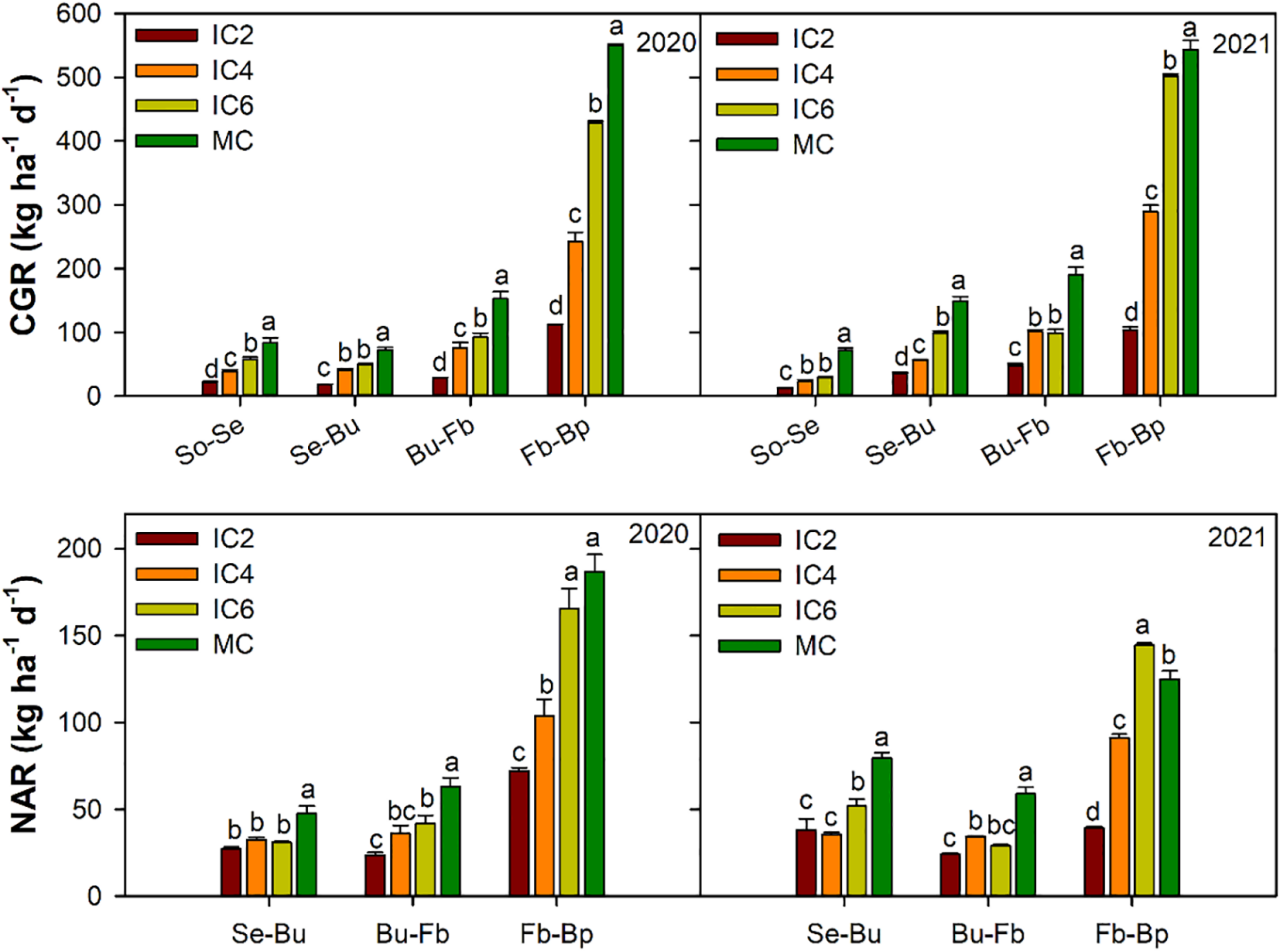
Crop growth rate (CGR) and net assimilation rate (NAR) at different period in 2020 and 2021. So-Se, Sowing to Seeding; Se-Bu, Seeding to Budding; Bu-Fb, Budding to Flowering–boll, Fb-Bp, Flowering and boll to Boll opening. IC2, IC4, and IC6 represent jujube intercropped with two, four, and six rows of cotton; MC, monoculture cotton. Different lowercase letters indicate significant differences among treatments at *p<* 0.05.

Compared to MC, the CGR under IC2, IC4, and IC6 treatments significantly decreased by approximately 79%, 54%, and 27% in 2020 and by 79%, 51%, and 24%in 2021, respectively (**Figure 8**). Similarly, the NAR significantly decreased by 59%, 42%, and 19% in 2020and by 61%, 39%, and 14% in 2021, respectively. Compared to IC6, CGR was significantly reduced by 71% and 37% in 2020 and by 73% and 36% in 2021 under IC2 and IC4 treatments, respectively; NAR was significantly reduced by 48% and 28% in 2020, by 55% and 29% in 2021, respectively. In addition, the CGR and NAR under IC2 were significantly lower than those under IC4 by 55% and 29% in 2020, by 57% and 37%in 2021, respectively.

### 3.5 Response of crop yield and LER to the row configuration

**Figure 9:**
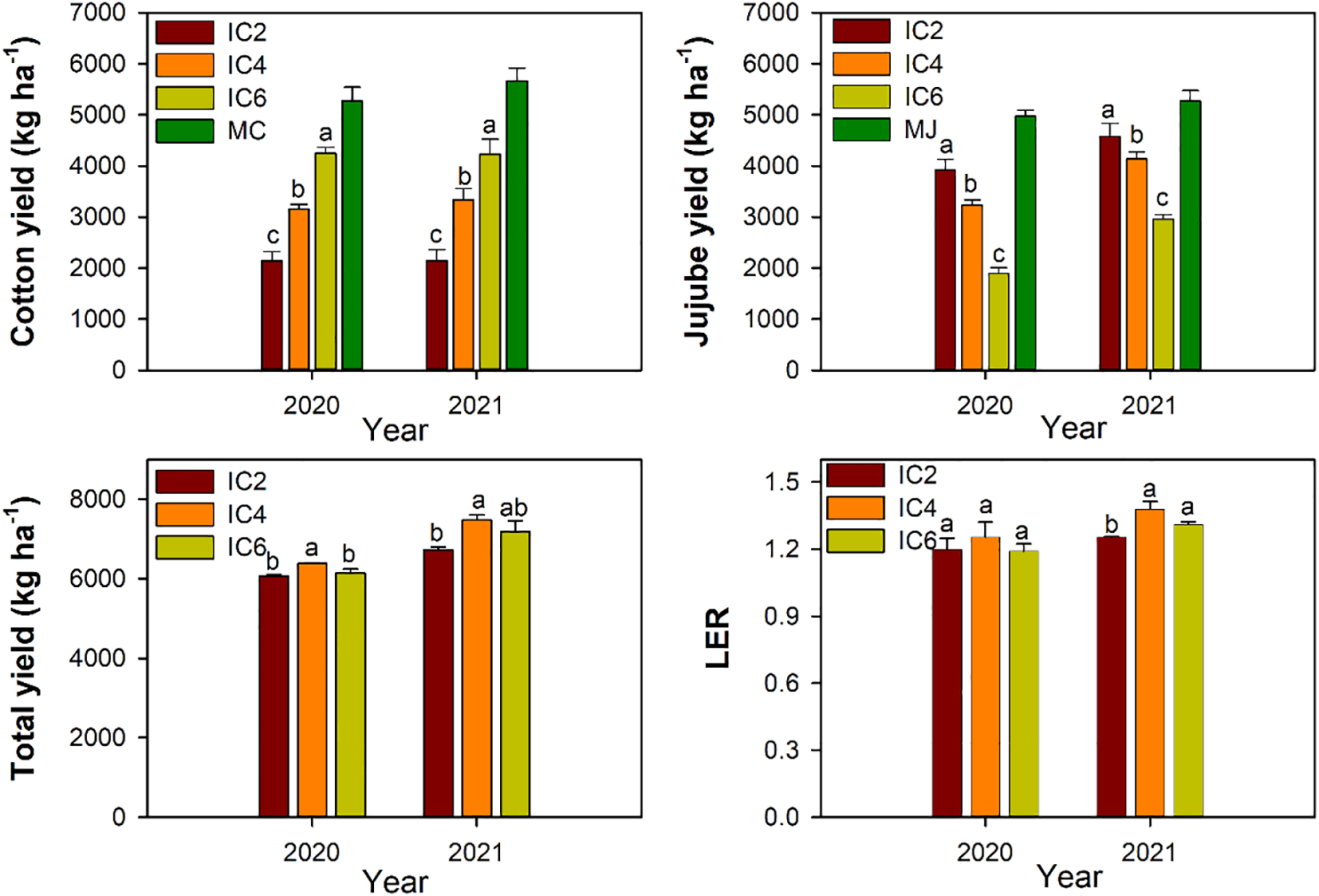
Yield and land equivalent ratio (LER) under different treatments in 2020 and 2021. IC2, IC4, and IC6 represent jujube intercropped with two, four, and six rows of cotton; MC, monoculture cotton. MJ, monoculture jujube. Different lowercase letters indicate significant differences among treatments at *p* < 0.05.

The yields of cotton and jujube in the intercropping system were lower than those in the corresponding monocropping treatments (**Figure 9**). cotton yield under IC2, IC4, and IC6 treatments were approximately 59%, 40%, and 19% lower in 2020, and were 62%, 41%, and 25% lower in 2021 compared to MC. Compared to MJ, jujube yield was 21%, 35%, and 62% lower in 2020, was12%, 22%, and 44% lower in 2021 under IC2, IC4, and IC6, respectively. Compared to IC2 and IC4, IC6 significantly increased cotton yield by 98% and 31%, while significantly decreased jujube yield by 43% and 34%, respectively. IC4 significantly increased cotton yield by 51%, but decreased jujube yield by 13% compared to IC2. The total yield under IC4 was increased by 5% and 4% in 2020 and by 11% and 4% in 2021 compared to the IC2 and IC4 treatments, respectively. IC4 and IC6 significantly increased the LER by 10% and 5%, compared to IC2 in 2021, respectively.

### 3.6 Cotton yield in relation to LAI, PAR, and growth characteristics

**Figure 10:**
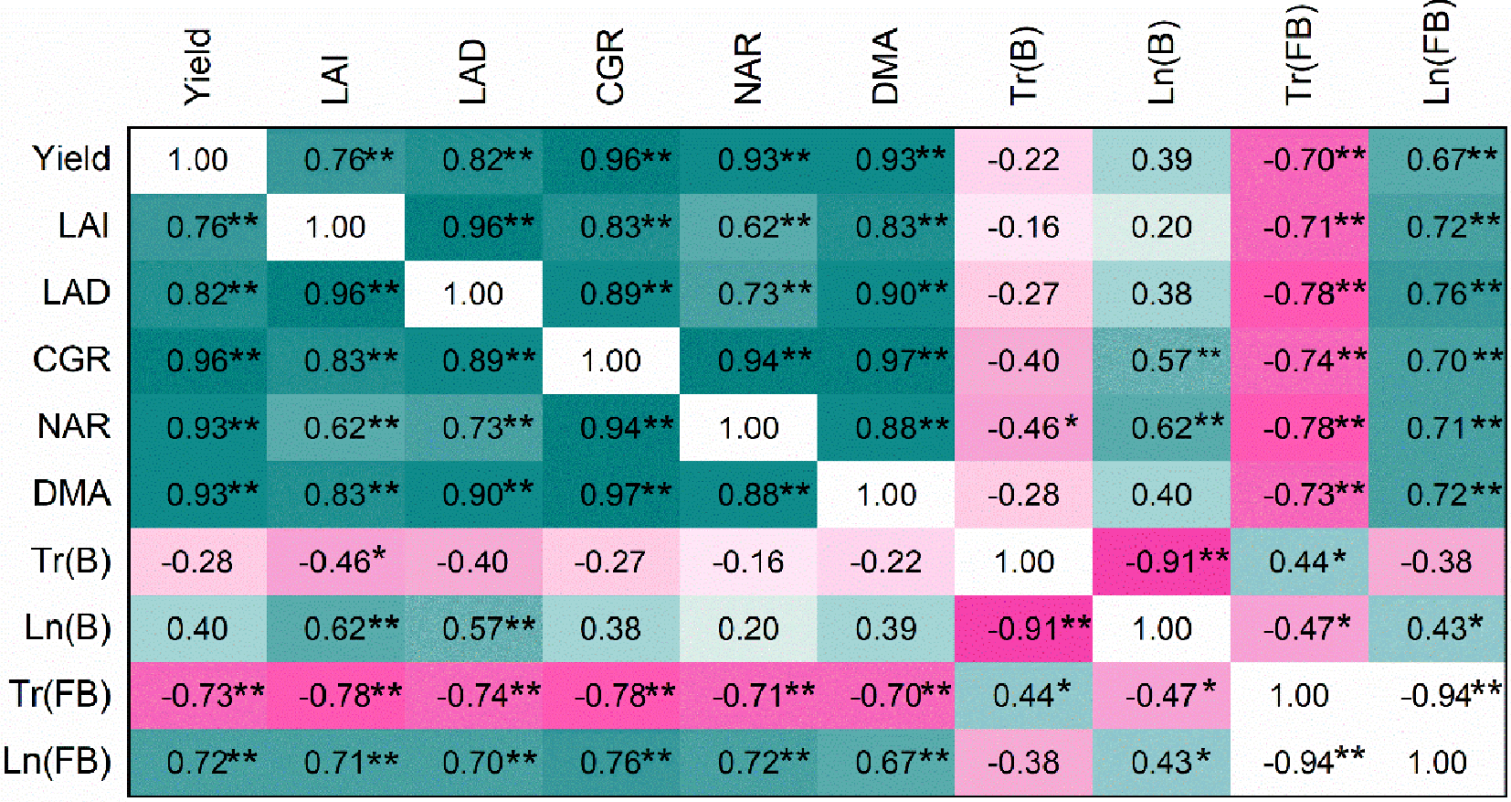
Relationships among cotton yield, growth characteristics, and photosynthetically active radiation. LAI, leaf area index; LAD, leaf area duration; CGR, crop growth rate; NAR, net assimilation rate; DMA, dry matter accumulation; Tr(B), Transmitted PAR rate at the boll stage; LN (B), Intercepted PAR rate at the boll stage; Tr (FB), Transmitted PAR rate at the flowering–boll stage; LN(B), Intercepted PAR rate at the flowering–boll stage. *p < 0.05; **p < 0.01.

**Figure 11:**
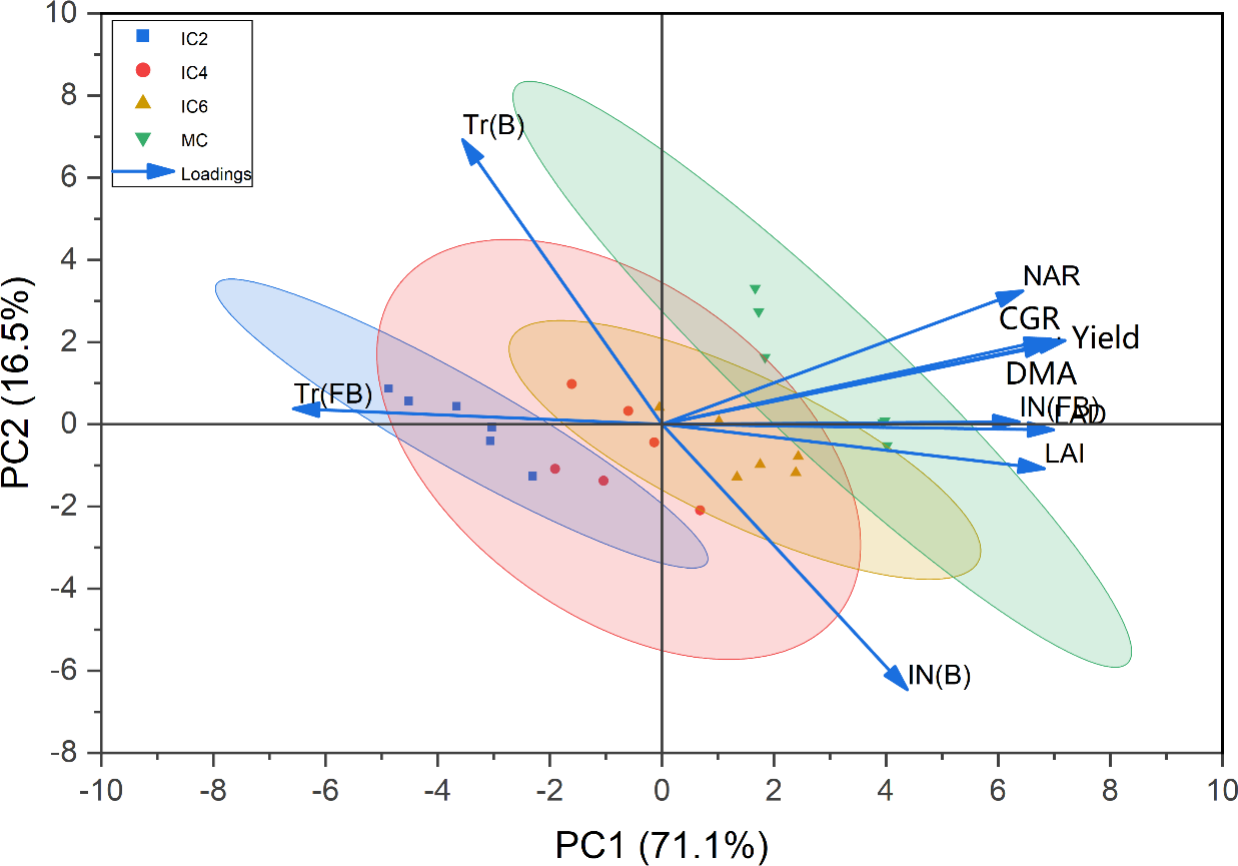
Principal component analysis among cotton yield, growth characteristics, and photosynthetically active radiation. LAI, leaf area index; LAD, leaf area duration; CGR, crop growth rate; NAR, net assimilation rate; DMA, dry matter accumulation; Tr (B), Transmitted PAR rate at the budding stage; LN (B), Intercepted PAR rate at the budding stage; Tr (FB), Transmitted PAR rate at the flowering–boll stage; LN (FB), Intercepted PAR rate at the flowering–boll stage.

Correlation analysis showed that cotton yield was significantly correlated with LAI, LAD, CGR, NAR, and PAR (Tr and IN at the flowering-boll stage) (**Figure 10**). PAR was significantly correlated with LAI and LAD; DRA and CGR were significantly correlated with PAR. Principal component analysis revealed that cotton yield was similarly related to LAI, PAR, and growth characteristics (**Figure 11**). 71.1% and 16.5% of the variability was explained by PC1 and PC2, respectively. LAI, Tr, DMA, and yield were positively correlated with PC1, while Tr was negatively correlated with PC1. These indicated that an appropriate increase in the number of cotton rows in jujube-cotton intercropping enhanced dry matter accumulation and yield by improving the LAI and increasing PAR.

## 4 Discussion

### 4.1 Effect of the row configuration on the LAI and LAD

LAI and LDA reflect the size of the crop’s photosynthetic organs and are key indicators of canopy community structure (Fang et al., 2019), which directly affects the crop’s photosynthetic production potential. The intensity of photosynthetic capacity is closely correlated with crop yield (Li et al., 2022a). Typically, the Leaf Area Index (LAI) presents a trend of initially increasing and then decreasing as the growth process advances (Li et al., 2022b). In the present study, LAI of cotton showed a tendency of increasing and then decreasing, reached the maximum value at the boll stage, consistent with the results of previous study (Li et al., 2022a). The LAI and LAD are susceptible to regulation through anthropogenic measures (e.g., planting density, tillage practices, fertilization, and irrigation), with planting density and row spacing configurations being the most significant factors affecting crop LAI (Kalogeropoulos et al., 2024). Optimized plant spacing configuration improves leaf spatial distribution, enhances photosynthetic efficiency, and boosts yield (Zuo et al., 2024). The present study found that cotton LAI and LAD under the intercropping system followed the pattern IC6 > IC4 > IC2, indicating that the LAI and LAD decreased with smaller populations and increased with larger populations. This study also found that the increase between IC2 and IC4 was greater than that between IC4 and IC6, this may be due to the fact that cotton plants and leaves rose with the increasing of planting density, but intraspecific competition occurred when the density became too high, which reduced the nutrients absorbed by individual plants, and reduced the cotton leaf area and thus weakened the increase in the LAI (Srinivasan et al., 2017).

### 4.2 Effect of row configuration on the PAR

The canopy structure significantly affects the photosynthetic productivity of cotton populations (Feng et al., 2016). LAI as an important indicator of crop canopy structure, affects light energy interception and DMA (Rogers et al., 2021). For cotton, light transmittance and interception rates directly influence the photosynthetic rate, thereby affecting the photosynthesis of lower and middle leaves and ultimately altering yield (Chapepa et al., 2020). In this study, the Tr of cotton showed the trend of IC6< IC4< IC2, and the IN showed the opposite trend. Tr and IN were significantly correlated with LAD and LAI, optimizing the cotton planting population improves the canopy structure, enhances the cotton population LAI, improves the PAR distribution within the canopy, and increases light energy use efficiency (Wu et al., 2023). In addition, Shading imposed by taller crops over shorter ones can become the dominant form of competition under conditions of sufficient water and high light intensity (Valladares et al., 2016). Taller-statured crops, benefiting from ample sunlight, exhibit robust photosynthesis and vigorous growth, typically developing extensive root systems, this enhances their capacity for water and nutrient uptake, thereby intensifying competitive pressure on shorter-statured crops (Iqbal et al., 2019). Conversely, shaded shorter crops experience reduced photosynthetic output, leading to diminished carbon allocation to their root systems. Increases in IN declined as number of cotton rows increased, suggesting that overly dense planting group shaded cotton leaves, thereby reducing the Tr (Hou et al., 2019; Niinemets, 2010; Valladares et al., 2016). Therefore, when cotton plant density is too low, the canopy intercepts less PAR, wasting light energy and reducing yield potential.

### 4.3 Effect of the row configuration on the DMA and yield

Crop population biomass is the direct product of photosynthesis, together with CGR reflect the functional capacity of photosynthetic organs and their production capacity, ultimately determining crop yield (Wang et al., 2020; Yin et al., 2017). The crop population size and row spacing configuration significantly affected CGR and NAR, thereby altering dry matter accumulation (Kuai et al., 2022). An appropriate planting density can optimize ventilation and light transmission within the population, improving its micro-meteorological environment (Yu et al., 2013), especially the enhancement of light energy utilization (Raza et al., 2019), thereby increase yield. Reasonable row spacing configuration regulates the cotton growing environment and shapes an efficient canopy, thereby promoting population dry matter accumulation and enhancing crop yield (Dong et al., 2025; Wu et al., 2023). In this study, DMA and CGR under IC6 were higher than those under IC4 and IC2and DMA were significantly correlated with LAI and PAR. This suggests that higher planting rows increased population dry matter accumulation by enhancing LAI and PAR interception, additionally, the increased planting density enhanced population dominance, which compensated for the reduced dry matter accumulation per plant (Dai et al., 2015). In addition, optimizing the row spacing configuration intercepts more light energy and stimulates stomatal conductance to open (Lu et al., 2023). Canopy light interception provides the energy foundation, while stomatal conductance acts as a key physiological regulatory valve controlling CO_2_ supply (Pang et al., 2023). Together, they influence and ultimately determine the photosynthetic efficiency at both the leaf and canopy levels, thereby promoting the conversion of carbon assimilation products into dry matter and increasing yield (Yao et al., 2017). Despite this, the growth rate increase exhibited a declining trend from IC2 to IC6, suggesting that further increases in planting rows may not lead to additional yield gains (Deng et al., 2012).

There was a significant effect between the cotton population size and row spacing configuration on yield components. Furthermore, an appropriate planting density facilitates establishing a rational population structure, enhancing dry matter accumulation, balancing bolls number and weight, and ultimately improving cotton yield (Chen et al., 2025). A major factor influencing cotton yield and yield components is PAR (Hu et al., 2021; Yang et al., 2022). In the present study, cotton yield under IC6 was significantly higher than that under IC4 and IC2 and was significantly correlated with LAI, PAR, CGR, and DMA. This further suggests that increasing cotton planting rows in the jujube-cotton intercropping system improves yield may by increasing the cotton LAI, enhancing the PAR, and promoting the accumulation and translocation of assimilated substances. However, in the jujube-cotton intercropping system, competition for soil nutrients and water also exists between the two plants (Wang et al., 2016). An increase in the yield of one crop inevitably decreases the yield of another (Zhang et al., 2014). The same trend was found in the present study, as the cotton planting rows increased, cotton yield increased but jujube yield decreased in the intercropping system. This may be due to the increase in the number of cotton populations in the intercropping system, which results in competition between cotton and jujube for resources such as light, water, and nutrients (Yin et al., 2020), increased competition may lead to earlier occurrence of light and water stress in plants, thereby affecting stomatal conductance, transpiration rate, and growth rate (Wang W. et al., 2022), and ultimately reducing the yield of jujube. In addition, this study also found that LER was greater under IC4 and IC6 than under IC2, but there was no difference between IC2 and IC6. Furthermore, the total yield under IC4 was higher than under IC2 and IC6. Therefore, the co-development of cotton and jujube requires coordination to optimize the total yield of the intercropping system. We recommend IC4 as the optimal treatment for the jujube-cotton intercropping system.

## 5 Conclusion

In this study, the LAI, PAR, CGR, dry matter accumulation, and yield were showed the pattern of IC6 > IC4 > IC2. Cotton yield was positively correlated with LAI, PAR, CGR, and dry matter accumulation. The LER under IC4 and IC6 were greater than that under IC2.The total yield under IC4 was higher than that under IC2 and IC6. Therefore, to synthesize the total yield of the intercropping system, four rows’ cotton planted between jujube trees is recommended for farmers to improve economic benefits. However, it is worth that water and fertilizer management and mechanized production in this system make it a challenge for production today. Further research should focus on integrated water and fertilizer management to reduce inputs while enhancing efficiency in the jujube-cotton intercropping system.

## Data Availability

The data analyzed in this study is subject to the following licenses/restrictions: The entire set of raw data presented in this study is available from the corresponding author upon request. Requests to access these datasets should be directed to guodongchen@taru.edu.cn.
