# Correction: Optimal row configuration in jujube-cotton intercropping systems increases cotton yield by enhancing growth characteristics and photosynthetically active radiation in arid region

**DOI:** 10.3389/fpls.2025.1717089

**Published:** 2025-10-29

**Authors:** Jinbin Wang, Peijuan Wang, Xiaofei Li, Zhengjun Cui, Ling Li, Qiang Hu, Hang Qiao, Wei Zhang, Sumei Wan, Guodong Chen

**Affiliations:** ^1^ College of Agriculture, Tarim University, Alar, China; ^2^ Key Laboratory of Genetic Improvement and Efficient Production for Specialty Crops in Arid Southern Xinjiang of Xinjiang Corps, Tarim University, Alar, China; ^3^ State Key Laboratory of Cotton Bio-breeding and Integrated Utilization, Institute of Cotton Research, Chinese Academy of Agricultural Sciences, Anyang, China; ^4^ College of Agriculture, Shihezi University, Shihezi, China

**Keywords:** jujube-cotton intercropping, four rows, photosynthetically active radiation, growth characteristics, total yield

There was a mistake in **Figures 8** and **9**, page 11 as published. **Figures 8** has lost its axis title, and **Figures 9** has lost its legend. The corrected **Figures 8** and **9** appears below.

**Figure 8:**
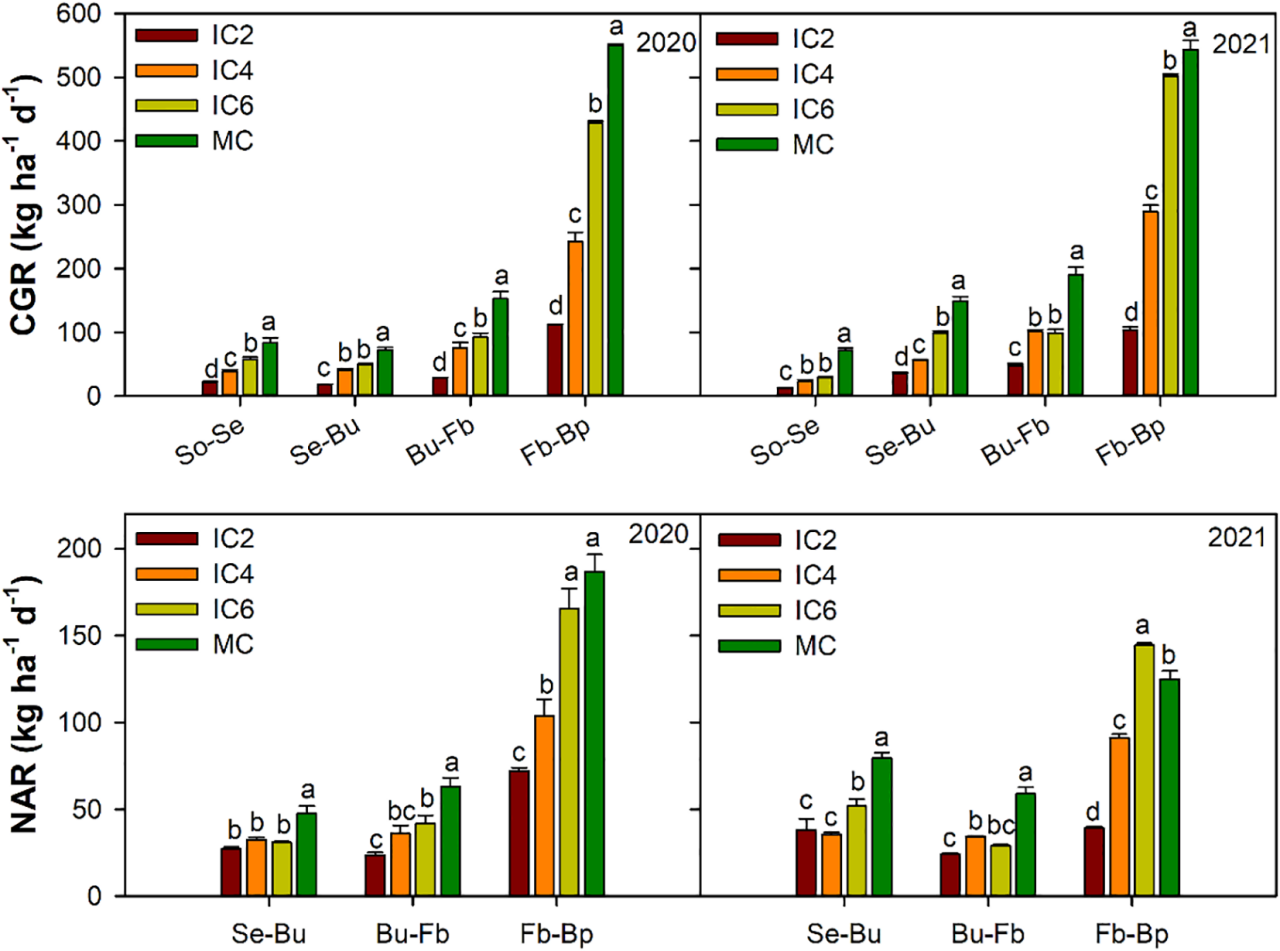
Crop growth rate (CGR) and net assimilation rate (NAR) at different period in 2020 and 2021. So-Se, Sowing to Seeding; Se-Bu, Seeding to Budding; Bu-Fb, Budding to Flowering -boll, Fb-Bp, Flowering and boll to Boll opening. IC2, IC4, and IC6 represent jujube intercropped with two, four, and six rows of cotton; MC, monoculture cotton. Different lowercase letters indicate significant differences among treatments at *p* < 0.05.

**Figure 9:**
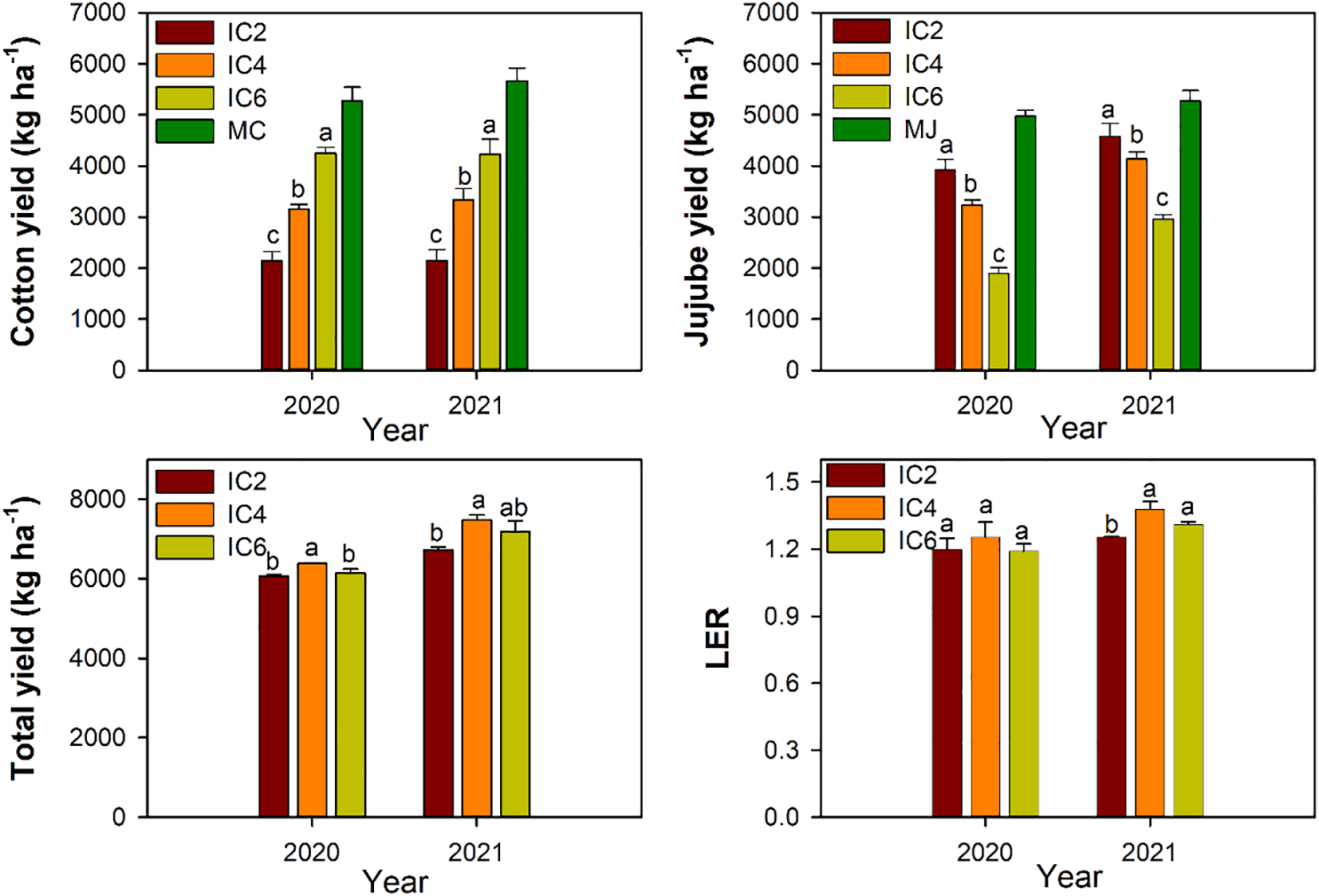
Yield and land equivalent ratio (LER) under different treatments in 2020 and 2021. IC2, IC4, and IC6 represent jujube intercropped with two, four, and six rows of cotton; MC, monoculture cotton. MJ, monoculture jujube. Different lowercase letters indicate significant differences among treatments at *p* < 0.05.

The original version of this article has been updated.

